# A novel unbalanced *de novo* translocation der(5)t(4;5)(q26;q21.1) in adult T-cell precursor lymphoblastic leukemia

**DOI:** 10.1186/1755-8166-5-21

**Published:** 2012-05-01

**Authors:** Eigil Kjeldsen, Anne Stidsholt Roug

**Affiliations:** 1 Cancer Cytogenetics Laboratory, Department of Hematology, Aarhus University Hospital, Tage-Hansensgade 2, DK-8000, Aarhus C, Denmark; 2 Laboratory of Immunohematology, Department of Hematology, Aarhus University Hospital, Tage-Hansensgade 2, DK-8000, Aarhus C, Denmark

**Keywords:** T-ALL, Unbalanced translocation, Oligonucleotide array CGH, der(5)t(4;5)

## Abstract

We here describe a novel unbalanced *de novo* translocation der(5)t(4;5)(q26;q21.1) in a 39-year-old male diagnosed with acute T-cell lymphoblastic leukemia. Bone marrow (BM) was massively infiltrated with 85 % highly proliferative polymorphic T-cell precursors. Immunologically, the malignant cells stained positive for CD7, CD34, intracytoplasmic CD3+, TdT + and negative for CD3 and CD5. G-banded chromosome analysis of BM cells showed the normal karyotype 46,XY[25] whereas BAC-based aCGH analysis revealed partial gain of 4q and partial loss of 5q. Multicolor karyotyping confirmed the presence of an unbalanced der(5)t(4;5) as the sole structural abnormality. Subsequent high-resolution oligonucleotide-based aCGH analysis showed that the der(5)t(4;5)(q26;q21.1) resulted in partial trisomy of 4q26qter (117,719,015-190,613,014) and partial monosomy of 5q21.1qter (100,425,442-180,857,866) and that there was no indication of any gene disruptions resulting from the breakages. Interphase FISH analysis using BAC-based specific probes for 4q26 and 5q21.1 confirmed the breakpoints and revealed approximately 80 % abnormal cells accordingly. At 4q26 the MIR1973 gene is located centromeric to the breakpoint in the copy number neutral region and the TRAM1L1 gene is located within the gained region. At 5q21.1 the genes ST8SIA4 and MIR548p are located centromeric to the breakpoint and no known genes up to approximately 1 Mb telomeric to the breakpoint in the copy number loss region. Interestingly, only the gene ST8SIA4 at 5q21.1 have been implicated in T-cell regulation as it encodes one of the key enzymes for polysialysation of surface proteins on dendritic cells which are important regulators for T-cell proliferation. The der(5)t(4;5) is thought to play a crucial role in the pathogenesis of acute T-ALL due to either gain of 4q, the loss of 5q, or deregulation of genes in proximity to the breakpoints.

## Background

Precursor T-lymphoblastic leukemia (T-ALL) accounts for approximately 25 % of patients with adult acute lymphoblastic leukemia and is a high-risk malignancy of lymphocytes committed to the T-cell lineage [1]. It is a heterogeneous disease and is diagnosed according to the expression of specific cytoplasmic or surface markers. The lymphoblasts are TdT positive and most often express CD3 and CD7. Moreover, variable expression of CD1a, CD2, CD4, CD5 CD7 and CD8 and HLA-DR is seen [2].

T-ALL has been associated with a normal karyotype in 30-45 % of cases [3,4]. Recurrent chromosomal translocations are reported in 25-50 % [5]. The most frequent abnormalities are del(6q), t(10;14). Translocations involving the TCR loci (14q11-TCRA/D and 7q34-TCRB) are found in about 35 % of T-ALL/LBL. A high percentage of cryptic abnormalities have been revealed by FISH mainly cryptic deletions at 9p21 and 1p32. Cryptic interstial deletion at 1p32 leading to SIL/TAL fusion gene is found in 9-30 % of childhood T-ALL. Normal karyotype and t(10;11)(q24;q11.2) are associated with better survival whereas the presence of any derivative chromosome is associated with poorer survival in childhood T-ALL [6].

In myeloid malignancies, the vast majority of recurrent chromosomal abnormalities are characterized either by fusion genes, as a consequence of reciprocal (balanced) translocations such as t(15;17) and t(8;21), or by sole genomic imbalances such as −5/del(5q), -7/del(7q) and trisomy 8. In contrast, acquired recurrent unbalanced translocations, which involve breakage and fusion of non-homologous chromosomes either at the centromeres or somewhere along their arms, are relatively rare in hematological malignancies. Unbalanced whole-arm translocations, such as der(1;7)(q10;q10), der(7;12)(q10;q10), der(9;18)(q10;q10) and der(3;10)(q10;q10) have been reported as a sole and recurrent anomalies, indicating that they could be the primary changes [7-10]. The most common of these are der(1;7)(q10;p10) which constitute a distinct entity of myeloid malignancies [8]. The clinical importance of other recurrent unbalanced whole-arm translocations remains unsettled. Reportedly, unbalanced whole-arm translocations are much more common than unbalanced translocations involving non-homologous chromosome arms. The most common is der(19)t(1;19)(q23;p13) that is strongly associated with precursor B-lymphoblastic leukemia (B-ALL) and can exist in a balanced and in an unbalanced form both resulting in a juxtaposition of PBX1 gene to TCF3 placing PBX1 under the transcriptional control of TCF3 on der(19). Regarding prognosis, however, there is some controversy whether the different forms affect clinical outcome [11,12].

There are only few cytogenetic prognostic markers described in T-ALL and the vast majority of these have been identified through the characterization of translocations. We describe here a novel unbalanced *de novo* translocation der(5)t(4;5) without additional chromosomal abnormalities in an adult diagnosed with T-ALL.

## Case presentation

### Clinical description

A 39-year-old male presented with a 5 weeks history of universal lymphadenopathy, fever and symptoms of extra hepatic cholestasis. Bone marrow (BM) examination was consistent with acute T-cell lymphoblastic leukemia (T-ALL) with an 85 % proportion of highly proliferative, polymorphic T-cell precursors with high nuclear cytoplasmic ratio, staining CD3-, CD5-, CD7+, CD34+, intracytoplasmic CD3+, and TdT+. PET-CT verified multi-nodal and widespread extra-nodal involvement of disease most prominent in the liver. Hematological examination included a total white blood cell count of 4.26 x10^9^/L, hemoglobin of 7.7 mmol/L and, platelets of 222 x10^9^/L.

G-banding of unstimulated cultured BM cells showed a normal male karyotype 46,XY[25]. To check for submicroscopic aberrations associated with T-ALL, FISH analysis for the following loci were performed: MLL, c-myc, bcr, TCF3 (all from DAKO, Denmark), SIL/TAL1, and T-cell receptor loci α/δ, β and γ (all from Abbott Molecular, Germany) of which all were negative.

The patient was initially treated with high dose steroid followed by induction therapy according to the high-risk arm of the NOPHO 2008 protocol. Minimal residual disease above the 5 % level was detected at day 29, which entailed repeated and intensified induction therapy. Nine and half months from initial diagnosis the patient died from serious infections after high dose chemotherapy while being in complete cytogenetic and hematological remission.

### Cytogenetic and molecular cytogenetic analyses

As part of a research program searching for submicroscopic genomic abnormalities in acute leukemia patients with normal G-banded karyotypes the patient was subjected to analysis by BAC-based aCGH analysis as described [13]. This type of aCGH analysis can detect genomic imbalances with a resolution about 1 Mb but can not detect balanced chromosomal structural rearrangements and revealed two large genomic imbalances: an appr. 72 Mb gain of chromosome 4 material from q26 to q35.2 and an appr. 80 Mb region with loss of chromosome 5 material from q21.1q35.3 (data not shown).

**Figure 1:**
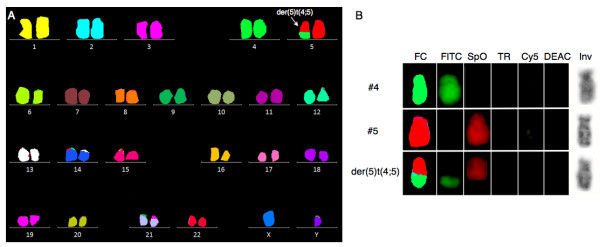
24-color mFISH karyotyping (24XCyte). **A.** A representative 24-color mFISH karyogram showing the unbalanced der(5)t(4;5) (arrow) as the sole cytogenetic abnormality. **B.** Single color gallery tool of normal chromosomes 4 and 5 and the unbalanced der(5)t(4;5). With this tool it is possible to show assigned false colors (FC) representing individual color schemes of labeled chromosomes arranged in its capture sequence (fluorescein isothiocyanate) FITC, (spectrum orange) SpO, (texas red) TR, (cyanine 5) Cy5, (7-Diethylaminocoumarin-3-carboxylic acid, succinimidyl ester) DEAC, together with an inverted grayscale image of the (4',6-diamidino-2-phenylindole) DAPI image (Inv). The top panel shows a normal chromosome 4 (#4) labeled with FITC, the middle panel shows a normal chromosome 5 (#5) labeled with SpO, and the bottom panel shows the unbalanced der(5)t(4;5) harboring labels from both chromosome 4 and 5. With the inverted gray scale image of der(5) it is not possible to identify the translocation.

To examine the cytogenetic basis for these findings, and to disclose other possible structural balanced abnormalities, we then performed 24-color karyotyping using 24XCyte human multicolor FISH (mFISH) probe kit according to manufacturer’s instructions (MetaSystems, Altlussheim, Germany) consisting of 24 different chromosome painting probes, each labeled with one of five fluorochromosomes or a unique combination thereof (combinatorial labeling). Image capture was done with an automated Zeiss Axio Imager.Z2 equipped with a CCD-camera (CoolCube1, MetaSystems) and appropriate filters using Isis software. Karyotyping was done using the 24-color mFISH upgrade package. Of 12 metaphases analyzed, 7 were abnormal all harboring the unbalanced translocation der(5)t(4;5) without additional structural chromosomal abnormalities (Figure 1). Re-analysis of the G-banding could not unequivocally identify the der(5)t(4;5), which also remained cryptic upon analysis of inverted gray scale image of the DAPI counterstain image channel of positively identified translocation positive chromosomes (Figure 1B).

**Figure 2:**
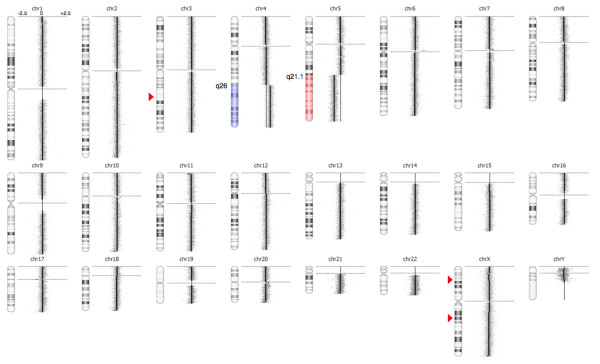
High-resolution array CGH reveals the unbalanced translocation der(5)t(4;5)(q26;q21.1) and three small copy number alterations. To the right of the individual ideograms microarray profiles of copy number gains and losses are depicted. Gain is indicated by blue color and loss is indicated by red color as an overlay on the ideogram. The log_2_ ratios for each chromosome are −2.5, 0, and +2.5 as illustrated for chromosome 1. Red triangles indicate small copy number aberrations that are not known CNVs at 3q22.3 (pos. 140,098,965-140,157,676), Xp22.1 (pos. 24,674,165-24,767,944) and Xq21.1 (78,433,612-78,518,546).

**Figure 3:**
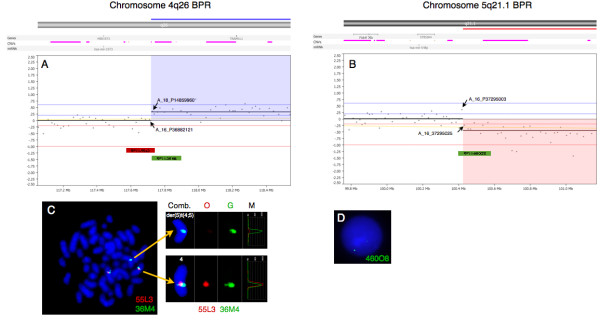
High-resolution array CGH reveals the breakpoints of the unbalanced translocation. Vertical blue lines indicate log_2_ ratios +0.24, +0.60 and 2.50 and red lines indicate log_2_ ratios −0.24, -1.00 and −2.50. At the top are indicated location of genes, CNV’s and miRNA. The X-axes at the bottom indicate chromosomal position. **A**. The break point at 4q26 was between the oligo-ID’s A_16_P36882121 (pos. 117,710,502) and A_18_P14859960 (pos. 117,727,528) (arrows). Blue shade indicates part of gained region. Location and size of the BAC probes RP11-55 L3 (red bar) and RP11-36 M4 (green bar) are indicated. **B.** The break point at 5q21.1 was between the oligo-ID’s A_16_P37295003 (pos. 100,418,842) and A_16_P37295025 (pos. 100,432,041). Red shade indicates part of lost region. Location and size of the BAC probe RP11-460O8 (green bar) is indicated. **C**. Left panel. Metaphase FISH using BAC probes RP11-55 L3 (red) and RP11-36 M4 (green) showing two fused signals (normal chromosome 4) and one green signal on der(5)t(4;5). Right panel. Single color gallery tool showing individual color schemes, orange (O) and green (G), and intensity measurements of each color channel (M). As can be seen in the der(5)t(4;5) panel there is a weak red signal and a strong green signal showing that the break point is within RP11-55 L3. **D.** FISH using RP11-460O8 (green) showing a nucleus with 1G1g pattern indicating that there is partial mono-allelic loss of the probe representing the break point.

To map the break point regions (BPR) at 4q26 and 5q21.1 at the gene level, an oligonucleotide aCGH analysis was performed using CytoChip Cancer 4x180K v2.0 (BlueGnome, Cambridge, UK) encompassing a 20 kb backbone with highest concentration of probes at 670 cancer genes. The analysis was done according to manufacturer’s instructions using 0,5 μg patient DNA. After hybridization, washing and drying the oligo array was scanned at 2,5 μm with GenePix 4400A microarray scanner. Initial analysis and normalization was done with BlueFuseMulti v2.6. For analysis and visualization normalized log2 probe signal values were imported into Nexus Copy Number software v. 6.1 (BioDiscovery, California, USA) and segmented using FASST2 segmentation algorithm with a minimum of 3 probes/segment. Regions of gain or loss contained within copy number variable regions (CNVs) were discarded. Reference genome was NCBI build 36.1 (hg18). As expected, we found the two large chromosomal imbalances: a partial gain of chromosome 4 from q26 to q35.2 (pos. 117,710,502-190,613,014) and a partial loss of chromosome 5 q21.1 (pos. 100,418,842-180,857,866) together with three minor copy number losses (Figure 2). The analysis indicated that the breakpoint at 4q26 was between the oligo-ID’s A_16_P36882121 (pos. 117,710,502) and A_18_P14859960 (pos. 117,727,528) and that the breakpoint at 5q21.1 was between the oligo-ID’s A_16_P37295003 (pos.100,418,842) and A_16_P37295025 (pos. 100,432,041) (Figure 3A and B). To verify the breakpoints, FISH analysis using BAC-based BlueFISH probes (BlueGnome,Cambridge, UK) specific for the BPR were performed. A dual-color FISH assay on metaphases with the probes RP11-55 L3 (red) and RP11-36 M4 (green) specific for 4q26 (Figure 3A) showed one fused signal at each of the two normal chromosomes 4 and one green signal at the der(5)t(4;5) (Figure 3C, left panel). Analyzing the relative red and green fluorochrome intensities along the chromosome axis on one of the normal chromosomes 4 and the der(5)t(4;5), using the single color gallery tool in Isis (MetaSystems; Altlussheim, Germany), revealed a small portion of the red-labeled probe RP11-55 L3 present at der(5)t(4;5) (Figure 3C, right panel). This result strongly indicates that the breakpoint is close to the one end of this probe and confirms the breakpoint as uncovered by the oligo-based aCGH analysis. The green-labeled FISH probe RP11-460O8 specific for 5q21.1 (Figure 3B) revealed that approximately 80 % of the nuclei had one strong green signal and one minor green signal (1G1g pattern) (Figure 3D) while a minority of cells had two equally strong signals (2G pattern). This result shows that RP11-460O8 includes the breakpoint at 5q21.1 and confirms the breakpoint identified by the oligo-based aCGH analysis. Lack of material precluded further FISH analysis.

The breakpoints for the unbalanced translocation at 4q26 and at 5q21.1 is outside any known genes whereas a couple of genes are located within 1,5 Mb of the break points (Figure 3A and B, top). The MIR1973 gene is located approximately 0,3 Mb centromeric for the breakpoint at 4q26 in the copy number neutral region and the TRAM1L1 gene is located approximately 1,4 Mb telomeric from the breakpoint within the gained region. At 5q21.1 the genes FAM174A, ST8SIA4 and MIR548p is located up to 0,4 Mb centromeric from the breakpoint and no known genes up to app. 1 Mb telomeric from the breakpoint. From these data we concluded that there is no apparent gene disruption and therefore it is unlikely that the unbalanced translocation has resulted in a fusion protein.

## Discussion

Cytogenetic studies are useful in the clinical management of leukemias and may also give clues to leukemogenesis as cytogenetic changes associated with leukemias can hint toward the genomic disorganization of malignant cells.

We described a patient with T-cell ALL with a novel unbalanced karyotype 46,XY,der(5)t(4;5)(q26;q21.1)[13]/46,XY[12]. To our knowledge the der(5)t(4;5) has not previously been described in the literature [14]. However, two cases of peripheral T-cell lymphoma with reciprocal translocations involving 4q26, t(4;16)(q26;p13) have been reported. In one of these cases extended analyses showed that the translocation resulted in rearrangement of interleukin 2 gene but this gene is located at pos. 123,59 Mb at 4q27 [15,16]. There are no reported cases with translocations involving 5q21 [14]. Isolated trisomy 4 was reported in 107 cases (2 ALL, 10 bi-lineage acute leukemia and the rest mostly myeloid leukemias) with no reports on partial trisomy 4q. Whole or partial monosomy 5 has been extensively reported in myeloid disorders and only in two cases of chronic lymphatic leukemia.

Unlike balanced reciprocal translocations, in which the genes that become deregulated and the functional consequences of the rearrangements can be readily identified through analysis of the breakpoint regions, most chromosomal imbalances have functional consequences that are unknown. This is mostly because the imbalances affect large genomic regions containing multiple genes and the fact that tumors often have numerous unbalanced chromosomal abnormalities. This degree of genetic complexity has hampered delineation of the roles of individual chromosomal gains and losses. Unbalanced translocations between non-homologous chromosome arms are most often part of complex karyotypes but rarely seen as sole chromosomal aberrations making such cases important for further studies. Although we did not clone the BPR or investigate the gene expression the molecular cytogenetic results provide important clues for further clinical and diagnostic investigations. Gain of 4q or loss of 5q could result in a gene dosage effect.

Alternatively, sequences close to the BPR could interfere with critical genes on either side of the break point as in the case for der(19)t(1;19) in B-ALL [11,12]. In our T-ALL patient we identified the BPR at 4q26 (pos. 117,710,502) with the genes MIR1973 and TRAM1L1 located on each side of the breakpoint, and the BPR at 5q21.1 (pos. 100,418,842) with the genes FAM174A, ST8SIA4 and MIR548p located centromeric to the break point. The function of these genes is mainly unknown, except for the gene ST8SIA4 that is one of the key enzymes of biosynthesis of polysialic acid (PSA). Interestingly, it was recently shown that polysialysation of surface proteins on dendritic cells influence their T-lymphocyte interactions and that removal of PSA from their surface promoted activation and proliferation of T-lymphocytes [17].

The unbalanced der(5)t(4;5) as described in our case may have arisen through three different mechanisms: 1) from a balanced t(4;5) with initial loss of der(4) and subsequent doubling of the remaining normal chromosome 4, 2) from an initial trisomy 4 followed by translocation and loss of der(4) or 3) from a translocation in G2 phase of the cell cycle, with the der(4) and der(5) ending up in different daughter cells followed by selective growth advantage. All three alternatives result in partial trisomy of 4q involving loci telomeric to MIR1973 and partial monosomy of 5q involving loci telomeric to ST8SIA4.

Irrespective of the mechanism and what genes that are affected, the unbalanced der(5)t(4;5) may confer proliferative and growth advantages that contribute to neoplastic progression.

## Conclusion

We have described a novel unbalanced translocation der(5)t(4;5)(q26;q21.1) as the sole cytogenetic abnormality in a patient with *de novo* T-ALL. It is thought to play a crucial role in the pathogenesis of acute T-cell lymphoblastic leukemia either because of the gain of 4q, the loss of 5q or deregulation of genes in proximity to the BPR, as no apparent gene disruption was uncovered. One explanation that this unbalanced translocation has not been described previously could be that it is very rare or more likely that it is underreported due to the translocation’s cryptic nature. Further investigations are necessary for clarification.

### Consent

Written informed consent was obtained from the patient before publication of this case report and any accompanying images. A copy of the written consent is available for review by the Editor-in-Chief of this journal.

## Abbreviations

aCGH: array comparative genomic hybridization; BAC: Bacterial artificial chromosomes; BM: Bone marrow; BPR: Break point region; B-ALL: Precursor B-lymphoblastic leukemia; CNV: Copy number variable regions; FISH: Fluorescence in situ hybridization; T-ALL: Precursor T-lymphoblastic leukemia; PSA: Polysialic acid.

## Competing interests

Anne Stidsholt Roug and Eigil Kjeldsen declare no competing interests.

## Authors' contributions

EK collected and analyzed cytogenetic and molecular cytogenetic data. ASR collected and summarized clinical data. EK and ASR wrote the paper. All authors read and approved the final manuscript.

## Authors information

We would like to thank biotechnologists Bente Madsen and Pia Kristensen for excellent technical assistance. The study was supported by the Danish Cancer Society.
